# Volatile profile of bee bread

**DOI:** 10.1038/s41598-024-57159-y

**Published:** 2024-03-22

**Authors:** Katarzyna Pokajewicz, Darya Lamaka, Nataliia Hudz, Leonora Adamchuk, Piotr Paweł Wieczorek

**Affiliations:** 1 Department of Analytical Chemistry, University of Opole, 45-052 Opole, Poland; 2 Department of Pharmacy and Ecological Chemistry, University of Opole, 45-052 Opole, Poland; 3 Department of Drug Technology and Biopharmaceutics, Danylo Halytsky Lviv National Medical University, Lviv, 79010 Ukraine; 4 Department of Standardization and Certification of Agricultural Products, National University of Life and Environmental Sciences of Ukraine, Heroiv Oborony Street 15, Kyiv, 03041 Ukraine; 5 Laboratory of Methods for Assessing the Quality and Safety of Beekeeping Products, National Science Center “PI Prokopovich Institute of Beekeeping”, Akademika Zabolotnoho Street 19, Kyiv, 03680 Ukraine

**Keywords:** Bee bread, Volatilome, GC-MS, SPME, Palynological analysis, Biochemistry, Chemistry

## Abstract

Bee bread is one of the least studied bee products. In this study, ten bee bread samples were characterized using palynology and HS–SPME–GC–MS (headspace solid-phase microextraction gas chromatography-mass spectrometry). In total, over one hundred different volatile components were identified, belonging to different chemical groups. Only ten common components were detected in all the samples. These volatiles were ethanol, methylene chloride, ethyl acetate, acetic acid, α-pinene, furfural, nonane, nonanal, *n*-hexane and isovaleric acid. Several other components were commonly shared among various bee bread samples. Over sixty detected compounds have not been previously reported in bee bread. The analysis required a mild extraction temperature of 40 °C, as higher temperatures resulted in the Maillard reaction, leading to the production of furfural. The profile of volatile compounds of the tested bee pollen samples was complex and varied. Some relationships have been shown between botanical origin and volatile organic compound profile.

## Introduction

Honey bees (*Apis mellifera* L.) produce valuable natural products, also known as bee products, including honey, beeswax, propolis, royal jelly, bee pollen, bee bread (BB), and bee venom. Honey is undoubtedly the main and most popular bee product. It is widely used for human consumption and is used as a natural sweetener in food and pharmaceutical products. Beeswax, the second most popular bee product, is used in the production of a variety of goods, such as candles, furniture polish, cosmetics, and pharmaceuticals. In addition to these applications, bee products have been used for centuries to treat a wide range of conditions, including wounds, infections, and chronic diseases. The practice of apitherapy dates back to ancient times and is based on the idea that bee products have healing properties. Currently, apitherapy is a part of complementary and integrative medicine in many countries^1^. In the last thirty years, it has become a subject of much scientific research^2^. Bee products have gained the interest of scientists, and numerous studies have exhibited different biological activities such as antimicrobial, antioxidant, and anti-inflammatory, thus having beneficial effects on human health^3–6^. The main obstacle to introducing bee products into a clinical routine as medicinal products is the variability of these natural products and the challenge of standardizing them^6–8^.

Bee bread is one of the least popular and studied bee products^1^. It is made by bees by mixing pollen, honey, and the secretion of bee digestive glands, then storing the whole in a hive in anaerobic conditions. Such prepared bee pollen grains undergo fermentation due to the presence of different microorganisms, mainly lactic acid bacteria. Both fermentation and digestive enzymatic biotransformations cause pollen and nectar to break down, forming a nutrient-rich food rich in carbohydrates, amino acids, unsaturated fatty acids, vitamins, and minerals^1,9,10^. These processes are needed to destroy the external layer of pollen exine made of resistant sporopollenin. The fermented bee pollen grains, known as bee bread are regarded as being more bioavailable than crude bee pollen, and contain more vitamin K as well as polyunsaturated fatty acids^9,11^. Their high nutritional value allows them to serve not only as the primary source of nutrients for the growth of bees, as it was naturally intended, but also makes them a valuable food source for human consumption. While essential nutrients are responsible for nutritional benefits, minor components such as different phytochemicals, including phenolic acid, anthocyanins, volatile compounds, and carotenoids, might evoke some beneficial biological functions. Some authors have claimed that pollen and bee bread possess numerous potential therapeutic activities, such as anti-tumor, anti-inflammatory, antimicrobial, and dyslipidemia-correcting effects^1,4,5,12–14^. These claims, however, have no solid evidence in the scientific literature, and more research is needed to support them. Bee bread and bee pollen are considered to be rich sources of antioxidants due to the high content of phenolic compounds. In fact, they are considered to be richer sources of antioxidants than honey^4,9,12,15^.

It can be assumed the chemical composition of bee bread can vary depending on the types of pollen and nectar collected by bees (thus on botanical and indirectly—geographical origin), the microbiota of the bees, and the duration of the fermentation process. Fermentation results in the production of many volatile compounds. Some of the volatile compounds found in bee bread include alcohols, aldehydes, esters, and ketones^16,17^. In addition to the volatile compounds produced by the fermentation process and other digestive biotransformations occurring in the stored grains, bee bread may also contain other volatile compounds of botanical origin—that are found in the pollen and nectar that the bees collect. These compounds can vary depending on the types of plants the bees visit, and they may include terpenoids, flavonoids, and other plant-derived compounds. These compounds contribute to the pleasant aroma of bee bread and may evoke some physiological effects when consumed. The BB volatilome is the profile of volatile organic compounds that are emitted by bee bread. Its investigation can provide important information about BB composition, flavor, and aroma, as well as safety and potential health effects. The volatile profile of bee bread has been hardly studied and is poorly understood. Previous studies have focused on honey, and to a lesser extent, propolis or unstored bee pollen. According to our best knowledge, there are only two articles in the literature that have explored the volatiles of *Apis mellifera* bee bread using solid phase microextraction from headspace (HS-SPME). The first study was conducted by Kaškoniene et al.^17^, who studied only one bee bread sample from Lithuania, along with honey samples of various floral origins. The second study was done by Starowicz and coworkers^16^, who characterized volatiles and sensory profiles of beeswax, bee bread, bee pollen, and honey from Poland. The authors of this study also analyzed only one sample of bee bread. One another related study was conducted by Montaser et al. who used gas chromatography coupled mass spectrometry (GC–MS) with headspace injection (HS) to analyze three bee bread samples of Egyptian origin.

As a result, the volatile profile of only few bread samples have been recorded in existing scientific literature. This is not sufficient to characterize this bee product, and further studies are needed to understand its composition in terms of volatile compounds. Therefore, the goal of our research was to analyze the volatile compounds emitted from various bee bread samples using solid phase microextraction from headspace (HS-SPME) and gas chromatography with mass spectrometry (GC–MS). The samples were collected in Eastern Europe and were identified according to their botanical origin.

## Methods

### Materials and reagents

Supelco alkane standard (C8-C40) and 1 cm long Supelco 50/30 μm StableFlex DVB/CAR/PDMS fiber was purchased at Merck (Darmstadt, Germany).

### Bee bread samples

**Figure 1:**
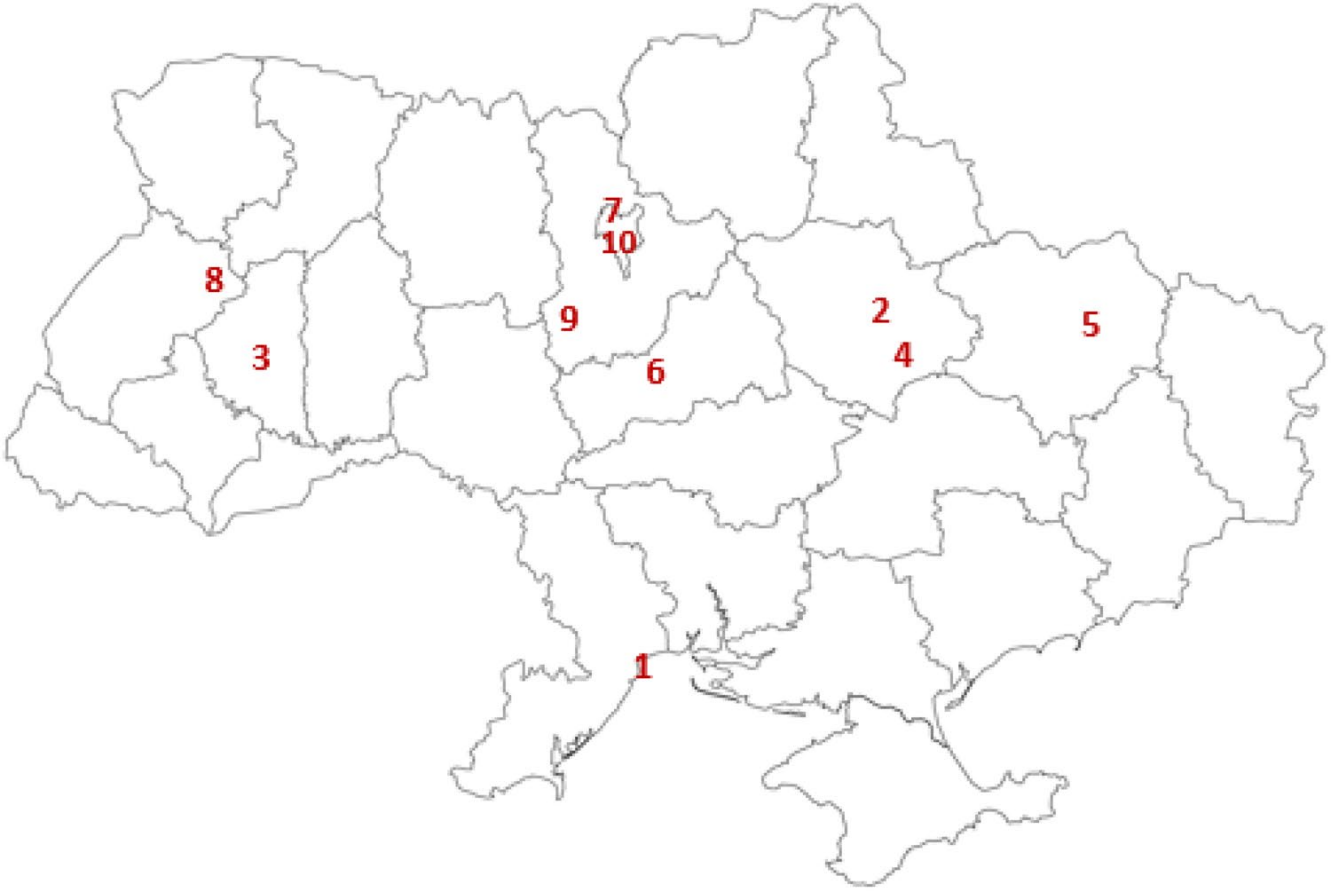
Ukraine. The place of origin of the studied samples.

Bee bread samples were taken from the *Apis mellifera* hives by professional beekeepers. Samples 1 to 10 were obtained in 2020 from different locations in Ukraine. Figure 1 indicates the origin place of the samples.

The samples were packed into polyethylene containers and kept in the freezer (minus 18 °C).

### Palynological analysis

The analyses were outsourced and performed at a professional laboratory specializing in the palynological analysis of bee products (HoneyLab, Puławy, Poland). To prepare microscopic preparations for palynological analysis, 10–40 mL of distilled water was added to each bee bread sample (from 5 to 18 g), depending on the weight of the sample. The mixture was shaken vigorously for 2 min, and shaking was repeated several times over the course of 6 h. The resulting homogeneous suspension was used to prepare a smear on the microscope slide. After drying the smear, the slide was covered with a coverslip with a drop of glycerol gelatin^18^. Palynological analysis of two smears was performed for each bee pollen sample, counting over 300 pollen grains and classifying them, if possible, to family, genus, species, or type of structure. Counting 300 pollen grains in one preparation allows one to obtain results that are representative of the entire sample^19^. The arithmetic mean was calculated from the two replicates, and its result was converted into the percentage of each type of pollen.

### Headspace solid phase microextraction

The volatile organic compounds were extracted and enriched using solid phase microextraction from the headspace (HS-SPME) of the bee bread sample. The mass of the sample was 2.0 g. The raw bee bread was placed in 20 mL N20 crimped vials, sealed with aluminum caps with silicone/PTFE septa. No solvent or salts were added. The samples were agitated and incubated for 15 min at a temperature of 40 °C. The following HS-SPME extraction lasted for 45 min (40 °C, constant agitation) and was performed using a manual holder and 1 cm long Supelco 50/30 μm StableFlex DVB/CAR/PDMS fiber (Merck, Darmstadt, Germany). After extraction, the SPME fiber was manually desorbed in the injector port of the gas chromatograph.

### Gas chromatography-mass spectrometry (GC–MS)

The analyses were performed using a Hewlett Packard gas chromatograph (HP 6890 series GC) with a 5973-mass detector (Agilent, Santa Clara, USA). The analytes were separated using ZB-5HT capillary column (5% diphenyl- and 95% dimethylpolysiloxane stationary phase, 30 m of length, an inner diameter of 0.32 mm, and a film thickness of 0.25 μm; Phenomenex Inc., Torrance, CA, USA). The carrier gas was helium with a constant flow of 2 ml/min. The oven was programmed as follows: initial 40 °C, kept for 5 min, then gradually increased by 3 °C/min to 180 °C, then changed by 15 °C min to 280 °C, and finally held for 1 min. The injector was set to 250 °C in spitless mode. After five minutes the injector was vented.

The GC–MS interface temperature was set to a temperature of 300 °C, the MS ion source temperature was 230 °C, and the scan range was 30–550 m/z.

### Analyte identification

The chromatograms were analyzed using MSD Chemstation (Agilent) and NIST MS Search software (version 2.7) (NIST, Maryland, USA). The data was analyzed using LRI created by the authors^20^ with the additional help of AMDIS software (NIST) in case of coelutions. The volatile analytes were identified by comparing the mass spectra data with the NIST 11 and NIST 14 libraries and by checking their retention index with literature values (using the Retentify tool and NIST Chemistry WebBook, SRD 69). The LRIs were calculated using an *n*-alkanes standard mixture from Supelco, which was also subjected to SMPE extraction before the analysis (Merck). The relative abundance was calculated by dividing the peak area of a particular analyte by the total peak area on the chromatogram (excluding time 0–3 min and peaks of silanes leaking from SPME fiber).

### Statistical analysis

All the analyses were carried out in triplicate, and the results were expressed as means. Standard deviations and coefficient of variation were calculated for each set of results. Due to the size of the dataset, this data is available in the supplement.

The statistical analyses were performed using MS Excel (Microsoft, Redmont, WA, USA) and the online platform MetaboAnalyst 6.0 (https://www.metaboanalyst.ca, Canada). The obtained data were analyzed using principal component analysis (PCA) and hierarchical cluster analysis (HCA, Ward method using Euclidean distance). These two classification techniques (PCA and HCA) were used to identify any clusters in the data and examine differences between the analyzed bee bread samples.

## Results and discussion

### Palynological analysis of bee bread

Overall, ten bee pollen samples were collected and subjected to analyses. The palynological analysis showed that the samples varied in terms of pollen species composition with different dominant plant species (Table 1). They also differed significantly in terms of their VOC profile.

**Table 1:** Palynological analysis of the tested bee bread samples.

Sample	The botanical origin of pollen [%]	Conclusion
BB1	***Sinapis*—mustard 23.4, *Medicago*—burclover 11.2***; Brassica napus*—rapeseed 9.0; *Trifolium* type—clover type 7.3; *Onobrychis*—sainfoin 5.8; *Lotus*—trefoil 5.4; *Papaver*—poppy 4.8; *Ranunculus*—buttercup 4.4; *Anthriscus* type—chervil type 3.9; *Prunus* type—plum type 3.6; *Echium*—echium 3.1; *Salix*—willow 3.0; *Thymus* type—thyme type 2.3; *Cornus*—dogwood 1.9; *Robinia*—locust 1.7; *Elaeagnus*—oleaster 1.5; *Melilotus*—melilot 1.5; *Achillea* type—yarrow type 1.2; *Cirsium* type—plume thistle type 0.7; other 4.4	A sample of bee bread varied in terms of pollen species composition. The largest percentage is mustard pollen (23%)
BB2	***Helianthus* type—sunflower type 61.1; *Zea mays*—maize 10.0**; *Centaurea jacea*—brown knapweed 5.4; *Cirsium* type—plume thistle type 3.2; *Melilotus*—melilot 2.5; *Thymus* type—thyme type 2.5; *Fagopyrum*—buckwheat 2.2; *Ranunculus*—buttercup 2.2; *Lotus*—trefoil 1.9; *Trifolium* type—clover type 1.6; *Ambrosia*—ragweed 1.3; *Anthriscus* type—chervil type 1.0; *Phacelia* 1.0; *Vicia*—vetch (broad bean) 1.0; *Centaurea scabiosa*—greater knapweed 0.4; *Tilia*—linden 0.3; other 2.3	Sunflower pollen dominates in the bee bread sample
BB3	***Medicago*—burclover 17.7; *Salix*—willow 16.5; *Fagopyrum*—buckwheat 12.8**; Papaver—poppy 8.1; *Brassica napus*—rapeseed 6.8; *Sinapis*—mustard 5.0; *Trifolium* type—clover type 4.6; Acer—maple 4.2; *Ranunculus*—buttercup 3.7; Thymus type—thyme type 3.5; *Solidago* type—goldenrod type 2.5; *Lotus*—trefoil 2.2; Rubus type—raspberry type 2.0; *Onobrychis*—sainfoin 1.7; *Echium*—echium 1.4; *Verbascum*—mullein 1.0; *Helianthus* type—sunflower type 0.9; *Cirsium* type—plume thistle type 0.7; *Anthriscus* type—chervil type 0.7; other 4.1	A sample of bee bread varied in terms of pollen species composition
BB4	***Melilotus*—melilot 41.8; A*mbrosia*—ragweed 20.8**; *Poaceae*—grasses 7.1; *Asteraceae*—Asteraceae 6.9; *Centaurea jacea*—brown knapweed 6.0; *Plantago*—plantain 3.0; *Cirsium* type—plume thistle type 3.0; *Onobrychis*—sainfoin 2.0; *Chenopodiaceae*—amaranth family 1.8; *Brassicaceae*—crucifers 0.9; *Polygonum*—knotweed 0.9; *Geranium*—geranium 0.8; *Phlox*—phlox 0.8; *Artemisia*—mugwort 0.8; *Vicia*—vetch (broad bean) 0.6; *Helianthus* type—sunflower type 0.1; other 2.7	Melilot pollen dominates in the bee bread sample
BB5	***Centaurea* sp.—knapweed (species unspecified) 41.8; *ambrosia*—ragweed 16.4; *Cirsium* type—plume thistle type 11.3**; *Artemisia*—mugwort 6.2; *Asteraceae* 5.3; *Medicago*—burclover 3.9; *Poaceae*—grasses 1.9; *Cytisus*—broom 1.6; *Chenopodiaceae*—amaranth family 1.5; *Trifolium pratense*—red clover 1.3; *Lotus*—trefoil 1.3; *Thymus* type—thyme type 1.2; *Cichorium* type—chicory type 0.7; *Zea mays*—maize 0.7; *Taraxacum* type—dandelion type 0.4; *Geranium*—geranium 0.4; *Elaeagnus*—oleaster 0.4; *Anthriscus* type—chervil type 0.4; other 3.0	The bee bread is dominated by the pollen of a plant belonging to the cornflower genus. Species not specified
BB6	***Tilia*—linden 21.7; *Brassicaceae*—crucifers 14.4**; *Rubiaceae*—bedstraw family 7.8; *Chenopodiaceae*—amaranth family 7.2; *Vicia*—vetch (broad bean) 5.4; *Trifolium* type—clover type 5.3; *Centaurea cyanus*—cornflower 4.4; *Helianthus type*—sunflower type 4.0; *Centaurea jacea*—brown knapweed 2.6; *Artemisia*—mugwort 2.5; *Lotus*—trefoil 2.5; *Melilotus*—melilot 2.5; *Achillea* type—yarrow type 2.3; *Heracleum* type—hogweed type 2.2; *Cirsium* type—plume thistle type 2.1; *Thymus* type—thyme type 1.9; *Zea mays*—maize 1.3; *Robinia*—locust 1.3; *Cornus*—dogwood 0.7; other 7.8	A sample of bee bread varied in terms of pollen species composition
BB7	***Salix*—willow 19.4; *Rubus* type—raspberry type 17.9; *Frangula*—buckthorn 15.5; *Prunus* type—plum type 13.4;** *Echium* 9.4; *Trifolium* type—clover type 4.1; *Achillea* type—yarrow type 2.7; *Lotus*—trefoil 2.3; *Brassicaceae*—cruciferous 2.1; *Rosaceae* 2.0; *Quercus*—oak 1.8; *Acer*—maples 1.7; *Vicia*—vetch (broad bean) 1.4; *Tilia*—linden 1.1; *Plantago*—plantain 1.1; *Caryophylaceae*—carnation family 0.5; other 3.8	A sample of bee bread varied in terms of pollen species composition
BB8	***Solidago* type—goldenrod type 49.5**; *Filipendula* 9.3; *Fagopyrum*—buckwheat 8.3; *Trifolium* type—clover type 5.6; *Papaver*—poppy 4.6; *Medicago*—burclover 4.2; *Sinapis*—mustard 3.6; *Frangula*—buckthorn 1.9; *Cynoglossum* 1.9; *Echium* 1.9; *Helianthus* type—sunflower type 1.6; *Centaurea jacea*—brown knapweed 1.4; *Centaurea cyanus*—cornflower 1.0; *Thymus* type—thyme type 1.0; *Anthriscus* type—chervil type 0.5; *Cirsium* type—plume thistle type 0.3; *Convolvulus*—bindweed 0.2; other 3.1	Goldenrod pollen dominates in the bee bread
BB9	***Centaurea* sp. (species unspecified) 52.3; *Cirsium* type—plume thistle type 19.0**; *Ambrosia*—ragweed 5.7; *Helianthus* type—sunflower type 5.3; *Chenopodiaceae*—amaranth family 3.7; *Ranunculus*—buttercup 2.4; *Taraxacum* type—dandelion type 2.3; *Echium* 1.7; *Echinops*—globe thistle 1.2; *Poaceae*—grasses 1.1; *Thymus* type—thyme type 0.9; *Frangula*—buckthorn 0.7; *Cichorium* type—chicory type 0.5; *Geranium*—geranium 0.1; *Polygonum*—knotweed 0.1; other 2.8	The bee bread is dominated by the pollen of a plant belonging to the Centaurea genus. Species not specified
BB10	***Brassica napus*—rapeseed 33.2; *Cirsium* type—plume thistle type 11.5**; *Anthriscus* type—chervil type 9.4; *Centaurea cyanus*—cornflower 9.1 *Centaurea jacea*—brown knapweed 6.1; *Helianthus* type—sunflower type 4.8; *Ambrosia*—ragweed 2.5; *Salix*—willow 2.2; *Zea mays*—maize 1.9 *Echium*—echium 1.8; *Trifolium* type—clover type 1.8; *Heracleum* typ—borscht type 1.6; *Cichorium* type—chicory type 1.5; *Medicago*—alfalfa 1.3; *Phacelia*—phacelia 1.3; *Thymus* type—thyme type 1.2; *Trifolium pratense*—red clover 1.0; *Fagopyrum*—buckwheat 0.9; *Centaurea scabiosa*—cornflower 0.6; other 6.1	Bee bread varied in terms of pollen species composition, with a slight dominance of rape pollen

### Analysis of volatile organic compounds of bee bread

The volatilomes of the studied samples were highly complex and varied. Tables 2, 3, and 4 in the manuscript, as well as Table S1 in the supplement present the result of the VOCs analysis. Altogether, 107 compounds were identified, belonging to different chemical groups including terpenoids (monoterpenes—13, oxygenated monoterpenes—10, sesquiterpenes—2), aldehydes (10), alcohols (7), ketones (6), carboxylic acids (11), esters (13), lactones (4), nitriles (3), sulfides (3) as well as alkanes (18), alkenes (3), and one representative of phenylpropanoid group. Six of them were also furan derivatives. Only ten components were detected in all of the samples: ethanol, ethylene chloride, ethyl acetate, acetic acid, α-pinene, furfural, nonane, nonanal, *n*-hexane, and isovaleric acid. Acetic acid, dimethyl disulfide, furfural, nonane, and nonanal were also observed in bee bread by Starowicz et al.^16^ and Kaškoniene et al.^17^. Isovaleric acid was detected by the first given authors only. However, due to strong adsorption on the (5%-phenyl)-methylpolysiloxane column, this analyte might be difficult to observe by the second authors if occurred at a lower level. Ethanol and dimethyl sulfide were observed by the latter authors only. Three of the above-discussed components, namely acetic acid, furfural, and nonanal, were also observed by Montaser et al. in *Trifolium* bee bread of Egyptian origin.

**Table 2:** Rough assessments of the most volatile compounds in the studied bee bread samples.

Retention time	Component	Sample									
		BB1	BB 2	BB 3	BB 4	BB 5	BB 6	BB 7	BB 8	BB 9	BB 10
0.79	Ethanol	S	S	S	S	S	S	S	S	S	M
0.82	Acetone	M	M	M	M	M		S	S	M	M
0.86	Dimethyl sulfide	S	S	M	d	tr	d	S	S		
0.89	Methylene chloride	B	B	B	M	M	M	B	B	B	B
1.02	*n-*hexane	M	M	M	S	S	S	S	B	B	B
1.07	Ethyl acetate	B	S	M	d	S	S	S	B	B	B
1.00–1.52	Acetic acid	M	M	M	M	B	B	B	B	B	B
1.41	1-penten-3-ol		S				S			S	
1.5	*n*-heptane	M	VB	M	VB	VB	VB		S	S	
1,92	Dimethyl disulfide				tr	tr	S				tr
2.21	Toluene				tr	tr	S			S	
2.72	Hexanal		M*		S*	S	M*	M*	M*	M*	S*
2.7–3.2	Butanoic acid						S	S	S	S	S

*S* small, *M* medium, *B* big, *VB* very big, *d* detected (due to coellution impossible to evaluate amount), *tr* traces.

*Octane coellution.

**Table 3:** The HS–SPME–GC–MS abundances [%] of volatile organic compounds from the studied bee bread samples.

	Compound	LRI_exp_	LRI_ref_	Relative abundance [%]										Compound group	Formerly detected in BB		
				BB1	BB2	BB3	BB4	BB5	BB6	BB7	BB8	BB9	BB10		L	P	E
1	Furfural	828	828	3.30	1.18	3.42	8.35	2.18	2.01	0.11	8.46	3.18	1.96	Aldehyde, furan der	y	y	n/y/n
2	(E)-2-hexenal	846	853		0.29	0.49	1.70		0.33					Aldehyde	n	n	n
3	Ethyl isovalerate	850	855								0.47	0.70	0.88	Ester	n	n	n
4	5-Cyano-1-pentene	855	858										0.79	Nitrile	n	n	n
5	Furfuryl alcohol	855	862								0.45			Alcohol, furan der	n	n	n/y/n
6	1-Hexanol	866	869			7.15								Alcohol	n	n	n
7	Isovaleric acid (= 3-methylbutanoic acid)	740–860	839–888	tr	0.35	4.56	4.59	9.55	2.41	0.43	13.48	6.04	6.42	Carboxylic acid	n	y	n
8	2-Methylbutanoic acid	na	861	0.14	0.17	9.65	2.27	10.99	tr	0.22	2.96		4.41	Carboxylic acid	n	y	n
9	2-Heptanone	890	891		0.39		2.07	2.74	1.98	0.14		2.07	0.69	Aldehyde	y	n	n
10	Nonane	899	900	3.54	0.23	9.45	6.33	3.67	2.47	0.33	2.74	2.15	1.27	Alkane	y	y	n
11	Heptanal	902	902		0.80				0.40	0.14	2.28	0.76		Aldehyde	n	n	n
12	2-Heptanol	904	899						0.22					Alcohol	n	n	n
13	Santolina triene	907	909										0.39	Monoterpene	n	n	n
14	2,6-Dimethylpyrazine or 2-acetylfuran	912	915/914	0.54						0.12				Na	n	n	n
15	γ-butyrolacton*	915	915	1.62		3.57	0.90		2.04	0.32	0.78	0.31	1.24	Lactone	n	y	n
16	Valeric acid	917	921								0.82			Carboxylic acid	n	n	n
17	*α-*Thujene	922	927	8.46	2.42				0.76		0.78	0.57	0.71	Monoterpene	n	n	n
18	*α-*Pinene	926	936	4.16	63.74	0.95	2.51	19.19	19.1	0.14	23.98	16.29	25.48	Monoterpene	n	n	n
19	Methyl hexanoate (= methyl caproate)	927	925	3.97		5.56	7.00							Ester	n	n	n
20	camphene	938	950		1.07				0.35			0.75		Monoterpene	n	n	n
21	*γ*-Valerolactone	953	954						0.22				0.20	Lactone	n	n	n
22	Benzaldehyde	958	962	1.55	0.34	tr	4.34		1.49	79.15	3.72		0.40	Aldehyde	y	y	n
23	Dimethyl trisulfide	959	971			3.23			1.22				1.24	Sulfides	y	n	n
24	Sabinene and β*-*Pinene coellution	966	973/978	14.98	8.28			1.89	3.73		2.26	2.72	4.40	Monoterpene	n	n	n
25	*α*-Methyl-γ-crotonolactone	980	989					3.7				2.22		Lactone	n	n	n
26	1-Octen-3-ol	983	980					2.81		0.67	0.46	2.34	0.88	Alcohol	n	n	n
27	Sulcatone	988	985	11.82	0.40	18.63		2.6	4.21	3.77	1.69	0.93	0.76	Ketone	n	n	n
28	*β*-Myrcene	990	989			1.10							1.06	Monoterpene	n	n	n
29	2-Pentylfuran*	992	990				2.11							Furan derivative	n	n	n
30	Decane	1000	1000	1.93	nd	5.76	0.88	tr	0.73	0.78	0.36	tr	0.47	Alkane	y	y	n
31	*α-*Phellandrene	1001	1004		0.23							8.00		Monoterpene	n	n	n
32	Ethyl hexanoate	1004	1003		0.47	0.96	1.71				1.10		3.09^y^	Ester	n	n	n
33	Octanal	1004	1002							0.54				Aldehyde	y	y	n
34	3-Carene	1003	1011					1.53	3.13			2.49		Monoterpene	n	n	n
35	*α-*Terpinene	1012	1017	4.5	2.75		8.89							Monoterpene	n	n	n
36	*p-*Cymene	1020	1024	3.91	2.15	1.27			3.36	0.21	1.57	3.00	3.69	Monoterpene	n	n	n
37	Limonene	1023	1029	3.97	2.96	4.43		3.99	13.66	0.32	1.33	11.61	8.25	Monoterpene	n	n	n
38	1,8-Cineol (= eucalyptol)	1022	1031				8.92							Monoterpenoid	n	n	n
39	caproic acid	1017–1029	980–1026				2.49	1.57						Carboxylic acid	y	y	n
40	Benzyl alcohol	1038	1036						tr	4.27				Alcohol	y	n	n
41	Benzeneacetaldehyde	1041	1045	1.40					0.87					Aldehyde	n	n	n
42	(E)*-β-*Ocimene	1048	1048	1.13		1.22			0.53	0.38		0.28	0.41	Monoterpene	n	n	n
43	*γ*-Terpinene	1054	1059	7.48	3.33				1.23		0.65	0.73	1.30	Monoterpenoid	n	n	n
44	Unidentified hydrocarbon	1056	na			3.81	6.95	1.80	0.74	0.30	0.5	0.84	1.16	Na	n	n	n
45	*cis*-linalool oxide (furanoid)	1069	1075						0.32		0.47			Monoterpenoid, furan der	n	n	n
46	3,5-Octadien-2-one	1072	1080		0.61				0.5		0.39	0.47	0.52	Ketone	n	y	n
47	terpinolene	1082	1086	1.13	0.59				0.27				0.21	Monoterpene	n	n	n
48	*trans-*linalool oxide*	1085	1083					1.33	1.36		0.96		0.61	Monoterpenoid, furan der	n	n	n
49	Methyl benzoate	1091	1094							0.45				Ester	n	n	n
50	2-Nonanone	1093	1093									0.34	0.37	Ketone	n	n	n
51	Undecane	1100	1100	6.28	0.10	6.8	2.26			0.71				Alkane	y	n	n
52	Linalool	1101	1099						1.25	tr				Monoterpenoid	n	n	n
53	Unidentified	1101	na				3.32				1.34	0.67	0.96	Na	n	n	n
54	Nonanal	1104	1103	3.63	0.44	2.53	3.19	1.70	2.27	0.86	2.12	1.76	1.20	Aldehyde	y	y	n
55	Isophorone	1114	1118						2.35	0.37				Ketone	n	n	n
56	Methyl octanoate	1127	1127	2.05	0.92	0.61	2.02	1.10	0.98	0.11	0.50		0.22	Ester	n	n	n
57	Benzyl nitrile	1140	1146							0.46			0.46	Nitrile	y	n	n
58	Ketoisophorone	1142	1142				0.92		0.42					Ketone	n	n	n
59	Terpinen-4-ol	1171	1177	2.07	1.15				0.66		0.98		0.98	Monoterpenoid	n	n	n
60	3,9-Epoxy-*p*-menth-1-ene (= dill ether)	1178	1178									1.50		Monoterpenoid, furan der	n	n	n
61	Nonanenitrile	1184	1182							0.19				Nitrile	n	n	n
62	*α*-Terpineol	1186	1189										0.15	Monoterpenoid	n	n	n
63	1-Dodecene	1191	1192									0.98		Alkene	n	n	n
64	Octanoic acid (= caprylic acid)	1198	1182						1.62		0.76			Carboxylic acid	n	n	n
65	ethyl octanoate	1200	1196		0.12						0.54		0.75	Ester	n	n	n
66	Dodecane	1200	1200				0.95		0.22	0.72		3.30		Alkane	y	y	n
67	Decanal	1205	1205						0.44	0.49	0.42	0.35	0.30	Aldehyde	y	n	n/y/n
68	3,6-Dimethyl undecane	1213	1210				0.92							Alkane	n	n	n
69	Methyl nonanoate	1226	1223						0.34					Ester	n	n	n
70	Nerol	1228	1228							0.17	0.84			Monoterpenoid	n	n	n
71	Bornyl acetate	1269	1283				1.34							Ester, monoterpenoid	n	n	n
72	2,6,11-Trimethyl dodecane*	1276	1275	1.57		2.73								Alkane	n	n	n
73	Nonanoic acid	1291	1275						0.5					Carboxylic acid	n	y	n/y/n
74	Tridecane	1297	1300				1.12		0.43	0.16				Alkane	y	n	n
75	Methyl decanoate	1324	1325						0.39		0.17			Ester	n	n	n
76	Eugenol	1356	1357							0.07				Phenylpropanoid	n	n	n
77	Tetradecane	1400	1400				0.51		0.26	0.09		0.37		Alkane	n	y	n
78	Caryophyllene	1406	1406	3.76	0.19			0.88	0.55	0.15	0.26	0.88	0.29	Sesquiterpene	n	n	n
79	*β-*Gurjunene	1418	1431		1.94				0.35		0.33	0.17	0.41	Sesquiterpene	n	n	n
80	Nerylacetone	1452	1455						0.19					Monoterpenoid der	n	n	n
81	2,3,7-Trimethyl decane*	1462	1466						0.18	0.09				Alkane	n	n	n
82	1-Pentadecene*	1492	1492						1.21		1.70		0.89	Alkene	n	n	n
83	Pentadecane	1500	1500						0.09	0.08	tr		tr	Alkane	n	n	n
84	Dihydroactinolide	1517	1537								0.56			Lactone	n	n	n

The most volatile components (0–3.5 min) were excluded. In the right columns, there is a mark if the analyte has been already detected in the bee bread so far.

Published compositions of bee bread of *L* Lithuanian origin^17^, *P* Polish origin^16^, *E* Egyptian origin^10^, *y* yes, *n* no, *nd* no data, *na* not applicable.

*Tentative identification (no available LRI literature value to confirm MS identification); tr—traces (0.05% or lower).

^x^Octanol coellution.

**Table 4:**
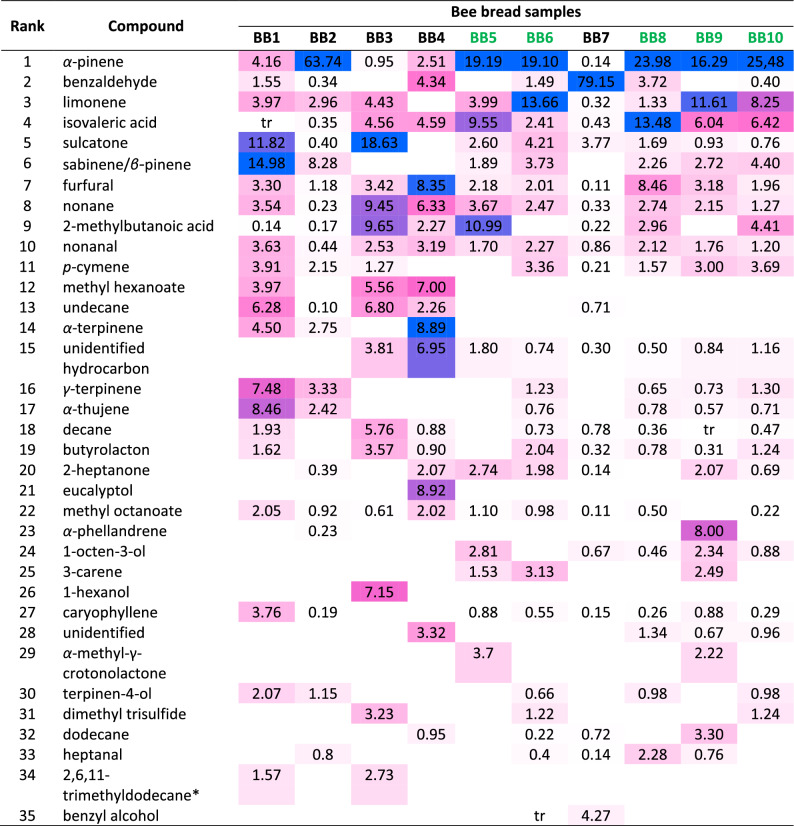
Top volatile components as % abundancies (peak of silanes and a front of the chromatogram excluded).

Acetic acid was found to be one of the most dominant components in this study, as well as in studies of cited above authors. It was also observed by Bakour et al.^21^ who determined acetic acid in the Romanian bee bread using HPLC–DAD at a level of 10.7 g/kg. Like ethanol, acetic acid derives as the product of biochemical transformations conducted by bee bread microorganisms. It is also abundant in the volatilome of bee pollen, which is the closest bee product to bee bread. Karabagias et al.^22^ found acetic acid to contribute to seven percent of the volatiles of Greek bee pollen samples. Prdun et al.^23^ analyzed multiple bee pollen samples of Croatian origin and found this carboxylic acid in only six of 21 analyzed bee pollen samples. Acetic acid is not generally found in the honey aroma, or it is found at much lower levels. For example, Kaškoniene et al.^17^ did not find acetic acid in any of the fifteen tested different honey samples of Lithuanian origin. Similarly, Makowicz et al.^24^ analyzed 15 samples of various honey from Poland and did not detect acetic acid in any of them. However, Starowicz et al.^16^ found acetic acid not only in bee bread and bee pollen but also in honey (at about four percent). Acetic acid is also present in the aroma of propolis. Kamatou et al.^25^ found it in South African propolis samples at varied concentrations, ranging from traces up to almost 62% in the headspace volatiles. Similarly, Cheng et al.^26^ found a significant fraction of acetic acid (ranging from 11 to 60%) in Chinese propolis volatiles.

We hypothesize that the presence of acetic acid and alcohol in bee bread is not connected to the botanical origin of bee pollen. The decrease in pH of stored bee pollen is caused by lactic and acetic acid fermentation, as well as alcoholic fermentation, which helps prevent the spoilage of BB. Bee pollen transforms BB through lactic acid fermentation primarily carried out by bacteria such as *Pseudomonas* spp., *Lactobacillus* spp., *Bacillus* spp., and yeasts such as *Saccharomyces* spp.^27^. Yeasts acquire energy via the conversion of various sugars into ethanol and carbon dioxide^28^**.** Lactic acid bacteria (LAB) constitute a diverse group of bacteria characterized by the production of lactic acid (LA) as the major metabolic end product of carbohydrate fermentation. LAB generate energy through substrate-level phosphorylation following two metabolic pathways for hexose fermentation, i.e., homofermentative and heterofermentative. The first pathway is based on glycolysis followed by the production of LA, whereas the second one, known as the pentose phosphate pathway, is characterized by the production of carbon dioxide, and ethanol or acetate in addition to LA^29^.

The presence of a large amount of methylene chloride is puzzling, but certain as the blank SPME in the same environment, using the same consumables, did not result in the appearance of a dichloromethane peak.

Chromatographic profiles of volatile organic compounds (VOCs) of studied bee bread samples were highly varied, both in terms of qualitative composition and the intensity of detected peaks. As mentioned earlier, only ten compounds were found in all of the tested samples, but several other components were also commonly shared among various BB samples. Namely, acetone, sulcatone (6-methyl-5-hepten-2-one), methyl octanoate, and limonene were identified in nine out of eleven samples, while 2-methylbutanoic acid, *p*-cymene, butyrolactone, decane, hexanal, *n*-heptane, and caryophyllene were detected in eight samples. Furthermore, benzaldehyde was indicated in seven samples. Other volatile compounds were found in fewer bee bread samples.

The results of VOC analyses are split into two separate tables (Tables 2 and 3) due to the fact there were big, mostly unresolved peaks at the beginning of the chromatogram, which could not be precisely integrated. Therefore, integration was inhibited from 0 to 3 min. Moreover, big peak areas of solvent front peaks would overwhelm the % abundancies, and further peak contributions would not be as readable (due to small % contributions).

To the best of our knowledge, over sixty detected compounds have not been previously reported in bee bread. Table 3 on the right side encompasses columns that indicate whether the analyte has been previously detected in this bee product. Among the most abundantly occurring volatiles in the studied samples that have not been already reported, there are α-pinene, α-thujene, γ-terpinene, limonene, *p*-cymene, α-terpinene, sabinene, β-pinene, and methyl hexanoate. Most of these are monoterpenes and their presence can be attributed to the plant material from which bee bread was made, as these are common specialized plant metabolites.

Apart from acetic acid and methylene chloride, the main components (ranked according to the sum of relative abundancies) were α-pinene, benzaldehyde, limonene, isovaleric acid, and sulcatone (Table 4). α-Pinene is a common plant monoterpene, most abundantly present in the essential oils of many coniferous tree species, but popular also in other plant species. It was present in all studied Ukrainian samples. Except for samples BB3 and BB7, it occurred at a significant level in the volatile fraction. In some samples (BB2, BB5, BB6, BB8-10) it was over 16%. The high abundance (~ 64%) of α-pinene in the volatile profile of the BB2 sample can be explained by its high abundance in the *Helianthus* essential oil. This BB sample contained 61% of *Helianthus* pollen. The essential oils of this plant contain monoterpene hydrocarbons, in particular α-pinene (49–59%), sabinene (2–17%), β-pinene (3–6%), and limonene (4–7%)^30^. BB8 sample also contained a high abundance of α-pinene that could be related to its content in essential oils in air-dried samples of aerial parts of *Solidago canadensis* L. (Asteraceae). Eight local invasive populations of *Solidago canadensis* L. from different countries contained α-pinene as a major component of essential oil (13–52%)^31^. Moreover, α-pinene (11–29%) was among the major components of the essential oils of four *Solidago* spp. of Lithuanian origin (*S. gigantea*, *S. canadensis*, *S. niederederi*, and *S. virgaurea*)^32^. We can relate its content and botanical origin due to its popularity in nature, therefore, we cannot use α-pinene as a specific marker of botanical origin for functional purposes.

The second-ranked component, benzaldehyde, is naturally occurring in many fruits and vegetables and has an aroma role with notes of almond, nuttiness, and stone fruit. It is one of the examples of volatile phenols, which are formed mainly in the shikimic acid pathway. It was not abundant in most of the samples except for sample BB7, which was distinct and contained benzaldehyde at almost 80% level. The palynological composition of this sample was varied and *Salix* pollen was the most dominant (about 20%), then; *Rubus* (18%), *Frangula* (15.5), and *Prunus* (13.4%) pollen grains were also abundant. Based on the obtained results, we do not see a correlation between the botanical origin of bee pollen and the presence of benzaldehyde. Benzaldehyde has already been detected in bee bread aroma by Kaškoniene et al., who reported it at a low level of 0.9%, and Starowicz et al.^16^, who found it at a higher level of 5.55%. It has been reported as an important constituent of bee products' aroma in numerous studies. For example, Kaškoniene et al.^17^ found it at contribution levels of 1.1–21.4%, and Starowicz et al.^16^ at abundance of 52.39% in multiflorous honey. It is also common in the volatile profile of bee pollen (0–40.3%)^16,22,23^, propolis (0.2–18.2%)^25,26^, and beeswax (4.3%)^16^.

Another common volatile compound occurring in the tested bee bread samples was limonene. Similar to α-pinene, it is plant monoterpene that is derived from methylerythritol phosphate (MEP) and mevalonate (MVA) pathways. We may assume that its presence is only due to the botanical origin of the bee bread samples. While it has not been reported so far in bee bread, it has been indicated in some other bee products. It is occasionally reported in honey volatiles^17,33,34^. Additionally, it can occur also in the volatilomes of bee pollen^22,35^ and propolis^26,36^. 11 samples of BP out of 14 from the Baltic region contained limonene^37^. In our study nine samples of BB contained limonene. We suppose that this monoterpene also is a nonspecific component of BP and BB^37^.

Isovaleric (3-methylbutanoic) and 2-methylbutanoic acids were among the most abundant volatile carboxylic acids in the tested BB samples, along with acetic and butanoic acids. Starowicz et al.^16^ observed 2/3-methylbutanoic acid in bee bread (1.2%) and multiflorous honey samples (14.9%). It is unclear whether the authors determined both 3-methylbutanoic and 2-methylbutanoic acids as coelution or presented the results as 2/3-methylbutanoic acid due to uncertainty in the identification of the correct isomer. Isovaleric acid has been also detected in the honey of cashew and marmeleiro origin^38,39^. 2- and 3-methylbutanoic acids have been indicated by Pasini et al.^34^ in six of ten studied buckwheat honey samples collected from Italy and Eastern Europe. The authors concluded that 3-methylbutanoic acid, along with a specific phenolic pattern, is a potential identifying characteristic of buckwheat honey. 2- and 3-methylbutanoic acids were also determined in the HS-SPME volatile profiles of linden, honeydew, and especially buckwheat honey samples^40^. These compounds were also assigned by the authors as buckwheat honey markers. Among our tested bee bread samples, only four of them—BB2, BB3, BB8, and BB10 had *Fagopyrum* spp. pollen identified at levels of 2.2, 12.8, 8.3, and 0.9%, respectively. BB3, which had a higher percentage of buckwheat pollen, did not have the higher contribution of the discussed carboxylic markers. Therefore, we may hypothesize that 2- and 3-methylbutanoic acids are not necessarily distinct markers of buckwheat origin. They might be present in the bee bread samples as by-products of microorganisms’ metabolism. Furthermore, out of 14 samples of bee pollen from the Baltic region, 11 contained 2-methylbutanoic acid^37^.

Another significant and frequent volatile component of bee bread is sulcatone (6-methyl-5-hepten-2-one). This hydrophobic unsaturated ketone can be found in all eukaryotes, ranging from yeast to humans. It is a commonly reported odor compound that is secreted with sweat by the human body and in different animals may play the role of alarm or attractant pheromone^41^. Sulcatone has been reported in numerous plants and essential oils from citronella, lemon grass, and palmarosa. It is a crucial component for the flavor profile of several fruits, including tomato, guava, and papaya^42^ where it is synthesized during carotenoid metabolism. Sulcatone has a flavor that can be described as a combination of apple, peach, and musty notes. Its presence enhances the sweet and fruity taste of numerous fruits^43,44^. It has been also detected in flower volatiles^45,46^. However, Ma et al. indicated that sulcatone does not act as a bee attractant but rather as a bee repellent. Nevertheless, it is a significant volatile and aroma contributor in our tested bee samples. It has been so far detected by Starowicz et al. in Polish bee bread (at a level of 4.4%) and bee pollen (0.7%). It has been also determined by Karabagias et al. in Greek bee pollen samples at levels of 6.1–7.9% and by Kaškoniene et al. in Lithuanian bee pollen at levels of 2.2–2.5%. Whether its origin is botanical or it is a product of bee metabolism or further biochemical processes occurring in bee bread, is unclear.

In our studies, nonanal was detected in all of the studied samples of bee bread. Nonanal was also identified as a component of bee bread by the previously cited authors^10,16,17^. Moreover, it was detected in all 14 tested bee pollen samples from the Baltic region^37^. It was suggested that some aldehydes and ketones are formed by the oxidation of fatty acids, particularly linoleic and linolenic^17^. According to Dąbrowska^47^, a significant increase in the concentration of nonanal and decanal in surface waters was recorded during the plant vegetation period. All of this can indicate that nonanal is a popular component of the plant kingdom and therefore, it is a nonspecific component of bee bread.

**Figure 2:**
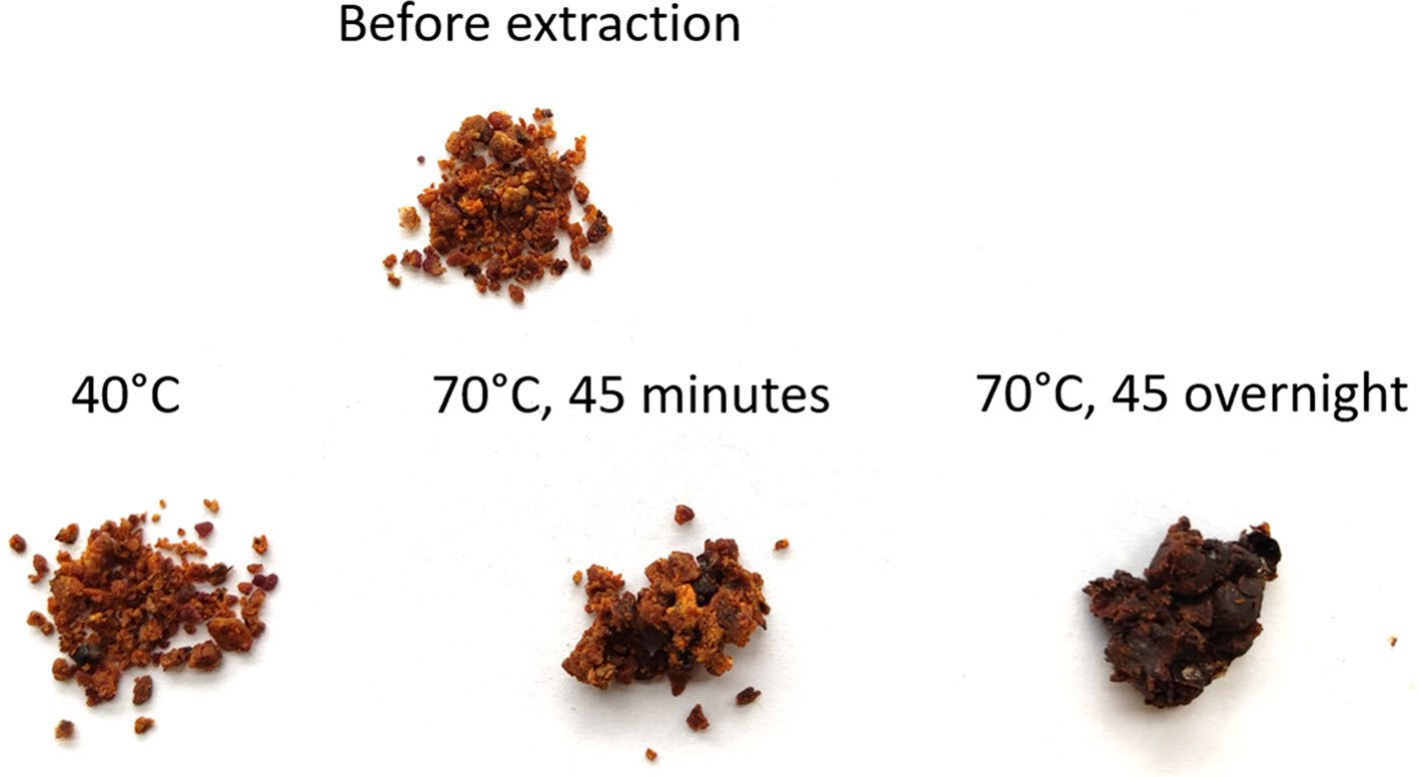
The influence of temperature on the appearance of bee bread (sample BB10).

Furfural has been detected in the volatile profile of all tested samples. Its presence was most probably caused by secondary reactions. Furfural is an important intermediate compound of the Maillard reaction of amino acids and reducing sugars to create brown melanoidins^48,49^. While heated, bee bread samples were gently “baked” and the furfural appearance had come from the Maillard reaction. When a higher temperature was used the bee bread was browned and stuck together (Fig. 2), in addition, furfural content raised significantly in the volatile fraction, from about 2% up to 44% (Table 5). When left overnight at 70 °C, the bee bread was burnt. Therefore, even though SPME extraction was more efficient in higher temperatures, higher peaks of analytes could be noticed or even new peaks detected, especially those with higher boiling points, the mild temperature of 40 °C was chosen for performing the extractions.

**Table 5:**
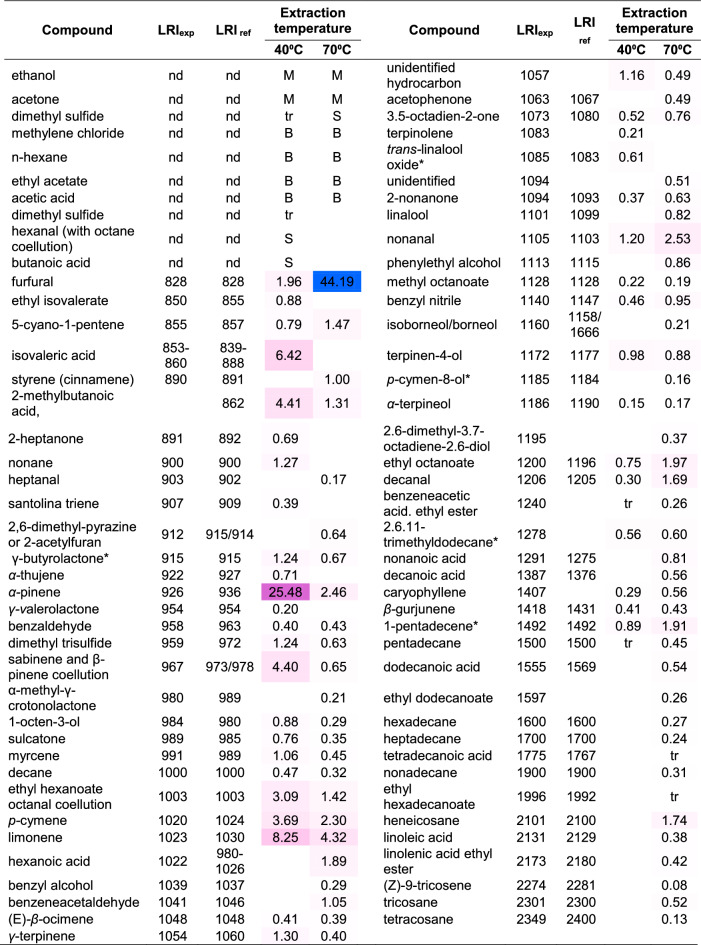
The influence of the temperature on the volatilome of bee bread (BB10); nd—not detected.

Apart from furfural, the volatile profile extracted at the higher temperature was richer in higher-boiling analytes such as long-chained hydrocarbons, fatty acids, and their esters (Table 5).

### Statistical analysis

**Figure 3:**
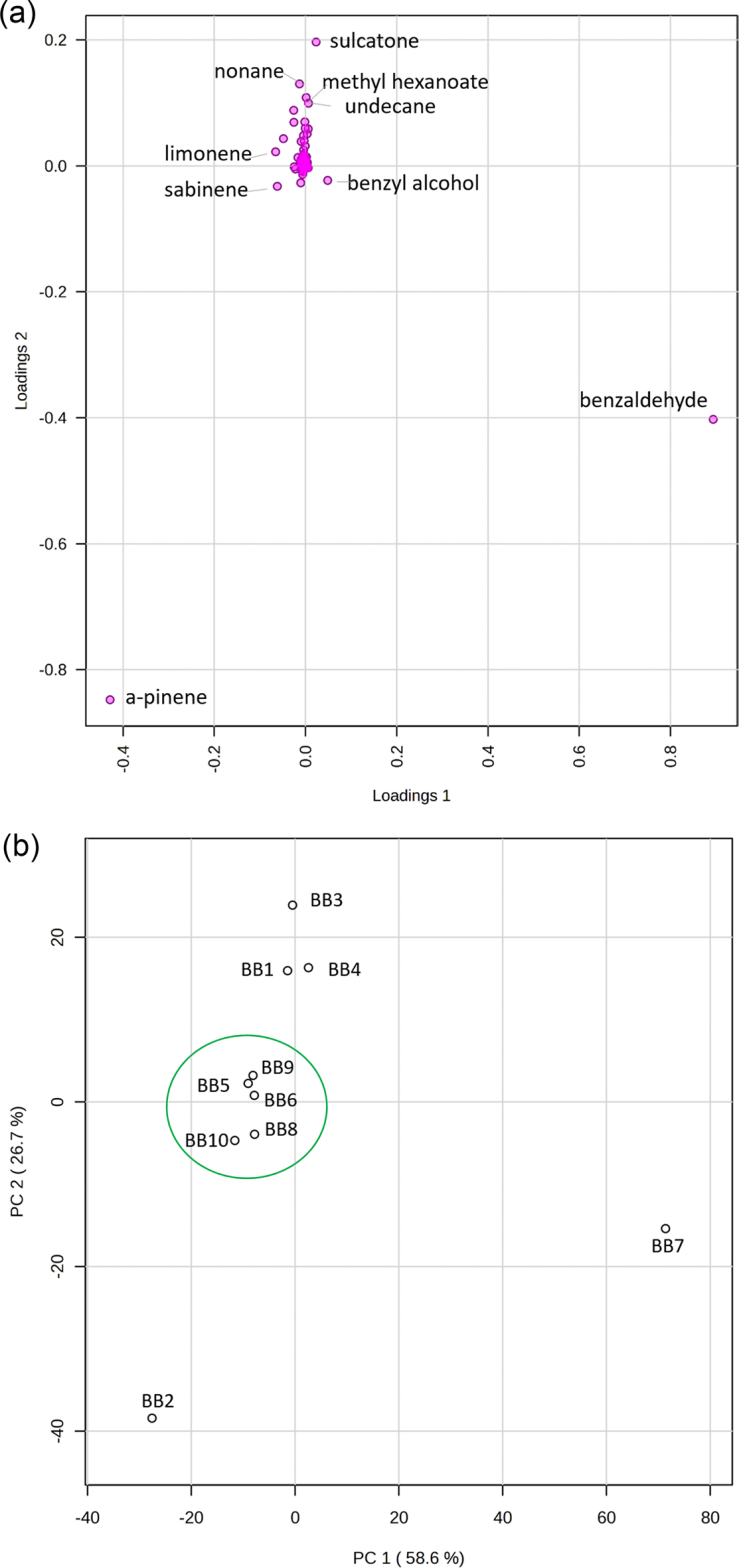
Loadings plot (**a**) and the score plot (**b**) of ten studied bee bread samples (BB1–BB10).

To discover natural groupings in the data and examine differences between the analyzed bee bread samples, statistical methods were implemented. Principal component analysis (PCA) was performed using mean-centered data, including 84 variables (detected analytes). The loadings and score plot are presented in Fig. 3a and b, respectively. The principal component (PC) 1 represents around 59%, and PC2 represents about 27% of the variance in the data. PC1 is mostly influenced by two chemical entities found in the bee bread volatilome: benzaldehyde, contributing positively to the PC1, and α-pinene, contributing negatively to PC1 (Fig. 3a). Regarding PC2, both benzaldehyde and α-pinene contribute negatively, while sulcatone, methyl hexanoate, undecane, and nonane contribute positively.

The most distinct sample is BB7, characterized by a completely different composition of the volatilome compared to the rest of the samples. This sample is positioned on the right in the PCA score plot due to its high abundance of benzaldehyde and only a minor content of α-pinene. Another apart sample is BB2. It is located in the lower-left quadrant of the plot due to the high abundance of α-pinene in the volatile profile—almost 64%, meaningfully more than the other samples. Samples BB5–6 and BB8–10 are clustered (as “green group”) in the center of the PCA plot (scores close to 0). These several samples are relatively similar, particularly when considering α-pinene at levels of 16–25%.

The samples B1, B3, and B4, located above the green cluster in the score plot, are characterized by smaller α-pinene content in the volatilome, and substantial amounts of methyl hexanoate, undecane, nonane, and additionally in the case of BB1 and BB3—sulcatone.

**Figure 4:**
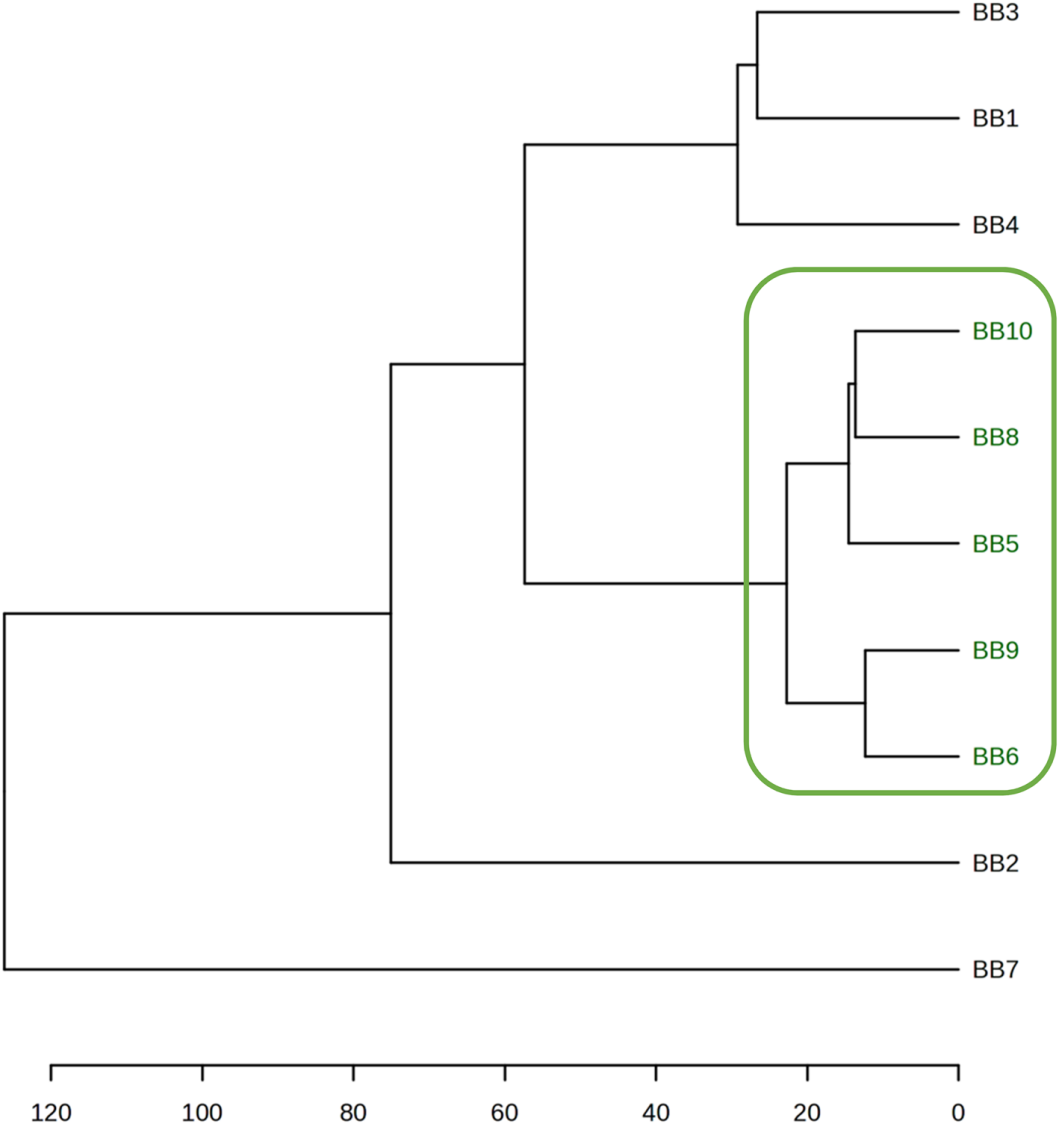
Dendrogram of the studied bee bread samples.

Figure 4 presents the relationships and grouping of the ten bee bread samples based on Euclidean distance and the Ward method. The results of hierarchical cluster analysis (HCA) are in line with the results of the PCA and confirm that the most distinct sample is BB7. It is mostly characterized by a high abundance of benzaldehyde. Other samples, such as BB1, BB2, BB4, BB6, and BB8 also contained some benzaldehyde. Based on the obtained results, we do not see a correlation between the botanical origin and the presence of benzaldehyde. As previously mentioned, BB7 contained pollen from *Prunus*, *Salix, Frangula*, and *Rubus*, while BB4 has neither of them but a lot of pollen from *Melilotus* and *Ambrosia*. The origin of benzaldehyde in bee products is poorly understood, and it is unclear if it is produced by plants or through other biochemical processes in the bee products or both. Benzaldehyde is widely present among volatile compounds and is probably phylogenetically one of the oldest compounds, as it is produced by more than 50% of plant families studied for their volatile profiles. Additionally, insects and non-insect arthropods also produce benzaldehyde^50^. It is also worth noting that benzaldehyde is used in beekeeping as a bee repellent for easy access to the hive to extract honey without being stung^51,52^.

The second distant sample, according to HCA, is BB2. This sample contained mainly *Helianthus* pollen grains and a higher fraction of α-pinene in the volatilome—about 64%, significantly more than the other studied objects.

The clusters formed in the dendrogram for samples BB5, BB6, and BB8–BB10 (green cluster) and the cluster for samples BB1, BB3, and BB4 are also in line with the result of the PCA.

The analysis of ten bee bread samples suggests some relationship between the volatiles existing and the botanical origin of the sample, as the indicated α-pinene resulting from *Helianthus*, *Solidago,* and *Ambrosia* pollen. However, this is a popular and non-specific monoterpene that exists in many other plant species. While attempts have been made to find volatile markers for botanical origin, the complex nature of bee product volatilome, the occurrence of these volatiles in multiple bee products of different origin, and the encountered natural variance may make it difficult or impossible to draw definitive conclusions. As discussed earlier, previous studies^34,40^ have identified 2- and 3-methylbutanoic acids as potential markers for buckwheat origin, but our results showed that these compounds were present in almost all tested bee bread samples of various origins. Therefore, any proposed marker would need to be validated using multiple samples of bee products of various botanical and geographical origins. According to Makowicz et al., high-performance thin layer chromatography (HPTLC) profiling of lipophilic extracts of honey may be more suitable for botanical origin authentication. The obtained chromatograms had the potential to create a type of identifier similar to a barcode, which could be used to distinguish between honey samples without requiring the identification of individual components^24,53^.

Nevertheless, our analyses have revealed the volatile components present in bee bread—one of the least studied and understood bee products. Many of the volatiles we detected had never been previously identified in bee bread before, greatly expanding our knowledge of its volatilome. Further research is needed to characterize the volatilomes of bee bread from different origins, explore potential relationships between its origin and composition, and gain a better understanding of biochemical processes occurring in the bee bread resulting in the presence of volatile components contributing to its pleasant aroma.

## Conclusions

In this study, ten bee bread samples from Eastern Europe were analyzed using solid phase microextraction from the headspace followed by analysis using gas chromatography coupled with mass spectrometry. The bee bread samples were of various botanical origins. A wide range of volatile components, comprising different chemical groups, were identified, over one hundred in total. The analyses revealed significant differences in the volatile profiles of studied bee bread samples. Despite the fact that most samples contained compounds from common chemical classes of identified compounds, such as hydrocarbons, terpenoids, alcohols, ketones, aldehydes, carboxylic acids, esters, lactones, nitriles, and sulfides, their qualitative and quantitative compositions differed from one another. Only ten components were found in all samples analyzed, including ethanol, ethylene chloride, ethyl acetate, acetic acid, α-pinene, furfural, nonane, nonanal, *n*-hexane, and isovaleric acid. Other components such as acetone, sulcatone, methyl octanoate, limonene, 2-methylbutanoic acid, *p*-cymene, butyrolactone, decane, and caryophyllene were commonly detected in various bee bread samples. The presence of furfural was enforced when using elevated temperature for SPME extraction, and mild temperature conditions were required to prevent alterations in the volatile profile. This may be explained by the fact that the Maillard occurs at elevated temperatures resulting in the production of furfural and brown melanoidins.

The obtained results allowed to characterize the volatile profiles of the various bee bread samples. However, it was impossible to pinpoint the exact volatile compounds that could be used as indicators of any botanical origin. Nevertheless, our results help to characterize the bee bread composition, one of the least studied bee bread products. Until our work, only the composition of a few samples has been studied and published, indicating the need for further and more in-depth characterization of this unique bee product. We discovered over sixty different components that have not been so far identified in bee bread.

### Supplementary Information

Supplementary Table S1.

## Data Availability

Data supporting the findings of this study are available within the article and its supplementary material. Raw data of this study is available from the corresponding author, upon request.

## Abbreviations

CAR: Carboxen
DVB: Divinylbenzene
GC: Gas chromatography
HCA: Hierarchical cluster analysis
HPTLC: High-performance thin layer chromatography
HS: Headspace
MS: Mass spectrometry
PCA: Principal component analysis
PDMS: Polydimethylsiloxane
PTFE: Polytetrafluoroethylene
SPME: Solid phase microextraction
VOC: Volatile organic compound
